# Transient Periictal Brain Imaging Abnormality in a Saudi Patient with Probable Celiac Disease Epilepsy and Occipital Calcification Syndrome

**DOI:** 10.1155/2019/5247961

**Published:** 2019-04-04

**Authors:** Soha Khan, Asma AlNajjar, Abdullah Alquaydheb, Shahpar Nahrir

**Affiliations:** King Saud Medical City, Riyadh, Saudi Arabia

## Abstract

Celiac disease epilepsy and occipital calcification (CEC) syndrome is a rare, emerging disease first described in 1992. To date, fewer than 200 cases have been reported worldwide. CEC syndrome is generally thought to be a genetic, noninherited, and ethnically and geographically restricted disease in Mediterranean countries. However, we report the first ever case of probable CEC in a Saudi patient. Furthermore, the patient manifested a magnitude of brain magnetic resonance imaging (MRI) signal abnormalities during the periictal period which, to the best of our knowledge, has never been described in CEC. The brain MRI revealed diffusion-weighted imaging (DWI) restriction with a concordant area of apparent diffusion coefficient (ADC) hypointensity around bilateral occipital area of calcification. An imbalance between the heightened energy demand during ictal phase of the seizure and unadjusted blood supply may have caused an electric pump failure and cytotoxic edema, which then led to DWI/ADC signal alteration.

## 1. Introduction

Celiac disease (CD), an autoimmune intestinal disease that customarily manifests as malabsorption syndrome, may also induce an autoimmune attack beyond the gastrointestinal tract [1, 2]. CD epilepsy and cerebral calcification (CEC) syndrome is a relatively novel entity first coined in the late 20th century. Initially, CEC was described only in Mediterranean countries and was considered a geographically restricted disease [3]. However, no environmental causation has so far been identified. Rather, a growing number of cases have emerged from different parts of the world in the last decade. We present the case of an elderly man of Saudi ethnicity with no antecedent malabsorption who presented with new onset occipital seizure and was unexpectedly found to have bilateral occipital calcification and a high titre of antitissue transglutaminase (TTG) antibody suggestive of probable CEC syndrome. This case underscores the need for suspecting latent CD in an elderly man of non-Mediterranean origin presenting with new-onset seizure, and transient magnetic resonance imaging (MRI) features suggestive of a periictal state.

## 2. Case Presentation

A 69-year-old Saudi man with diabetes mellitus was admitted to the Neurology Department with an unremitting headache lasting 5 days, episodic confusion, and visual disturbances. According to his family, the headache started gradually over the left side of his head and then became holocephalic and moderate to severe in intensity. The patient reported feelings of nausea and 2 episodes of vomiting. Moreover, his family stated the patient was often seen “bumping” into surrounding objects while ambulating. The patient reported experiencing some visual disturbance during this period. The patient's family felt he appeared confused at times and was not responding to his surroundings. He had no clear history of seizure, according to the family, and his past medical history was unremarkable apart from diabetes. He had sustained a minor head trauma 3 years prior with no concussion; however, his scan was reported to have shown “scattered areas of bleeding” in his brain. He otherwise maintained a healthy life and never required a hospital visit for any medical issues. His family reported the patient had normal cognitive function, especially as someone who ran his own business.

The patient was lethargic upon arrival to the accident and emergency department. Given the apparent risk of airway obstruction, the emergency physician intubated the patient. On general examination, we found no facial phakomas. While the patient was sedated, we found tonic eye deviation with nystagmoid-like eye movement and subtle myoclonic jerks of the distal limb suggestive of subclinical seizures. He was given an intravenous (IV) loading dose of phenytoin in addition to a midazolam infusion. Despite this, he sustained several clinical seizures in the subsequent days. Therefore, he required further titration of midazolam infusion (up to 14 mg/hr) and IV levetiracetam was added to optimize the antiepileptic coverage. His electroencephalogram was obtained postictal. His brain MRI was obtained 1 day following suspected subclinical seizures. Laboratory investigations showed peripehral blood cell count, haemoglobin, renal and liver function within reference range, and his blood glucose was elevated (14.7 mmol/L/264mg/dl).

Moreover, the results of his thyroid function test and his parathyroid hormone and serum vitamin B12 levels were normal. His serum folate level was not available. The screening tests for Hepatitis B antigen, Hepatitis C virus, and human immunodeficiency virus antigens and antibodies were all negative. His anti-TTG immunoglobulin A (IgA) titre was high at 35 U (reference range is up to 20 U), and the screen for antiendomysial antibody was negative, and his anti-Gliadin IgA antibody results were within reference range. Cerebrospinal fluid (CSF) study showed a WBC of 1, red blood cell count of 1, protein level of 36mg/dL, and a glucose level of 7.6mmol/L. The results of the CSF tuberculosis and herpes simplex virus 1 and 2 polymerase chain reaction were negative. We performed a duodenal endoscopic biopsy, but the specimen was not prepared properly; therefore, histopathological examination was suboptimal. We did note, however, increased intraepithelial lymphocytes with normal villous architecture.

**Figure 1:**
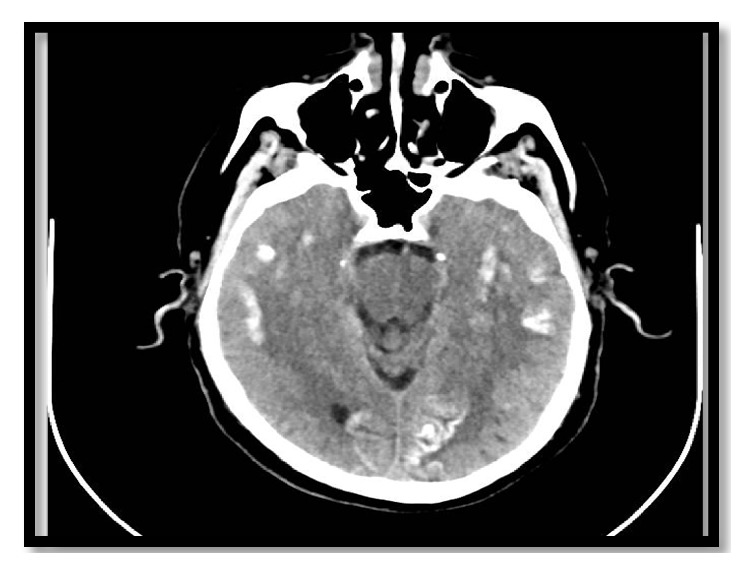
Plain CT of the brain at presentation to the hospital showing patchy and gyriform calcification in bilateral temporoparietal occipital corticosubcortical areas. Abbreviation: CT, computed tomography.

**Figure 2:**
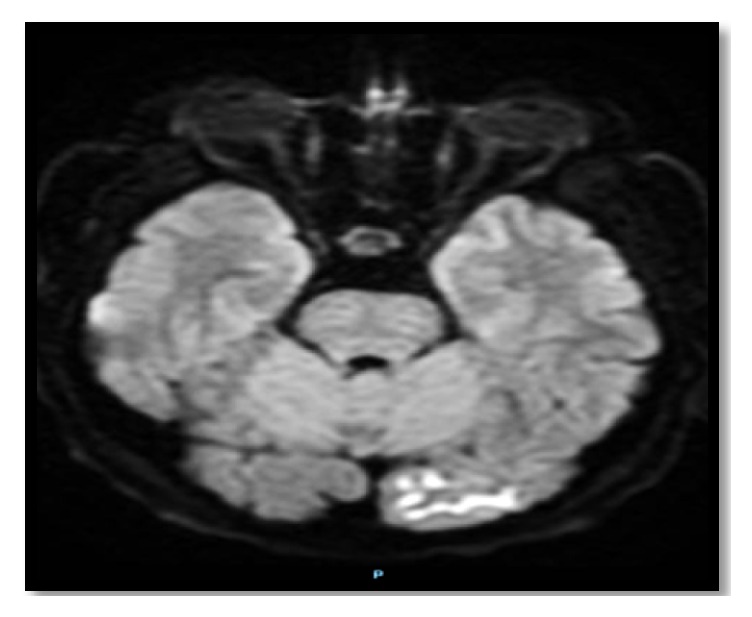
Periictal brain MRI DWI showing gyriform hyperintense signal over the left occipital cortex. Abbreviations: DWI, diffusion-weighted image; MRI, magnetic resonance imaging.

**Figure 3:**
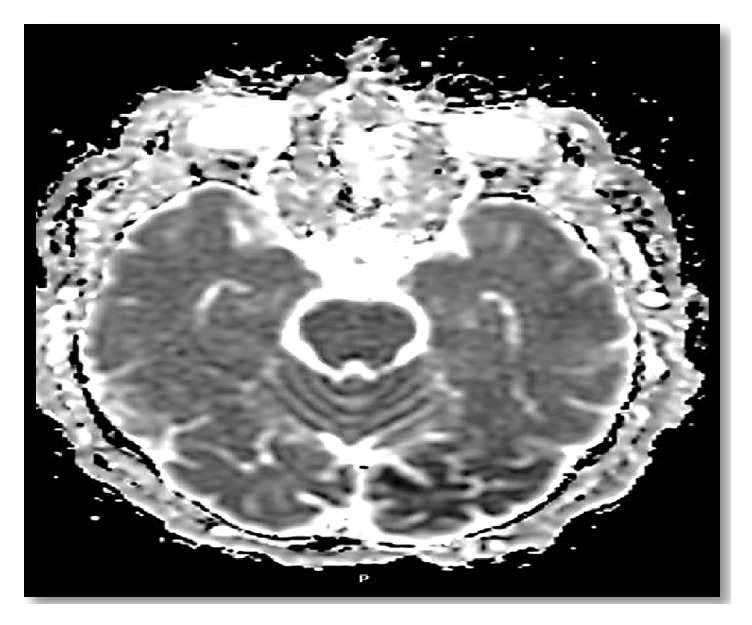
Periictal brain MRI ADC mapping showing bilateral occipital hypointense signal alteration (left>right). Abbreviations: ADC, apparent diffusion coefficient; MRI, magnetic resonance imaging.

**Figure 4:**
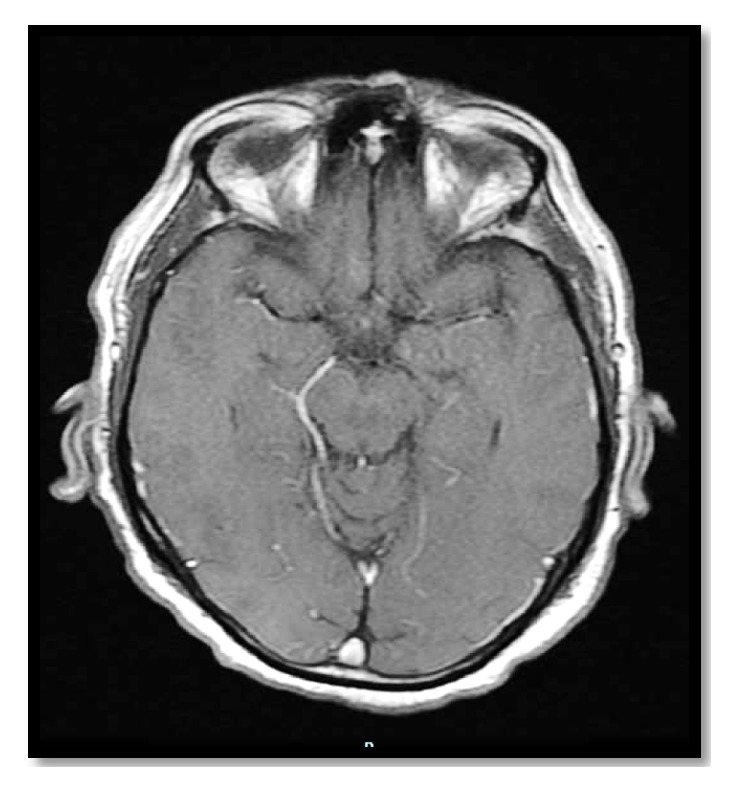
Periictal brain MRI with contrast showing no corresponding area of enhancement. Abbreviation: MRI, magnetic resonance imaging.

**Figure 5:**
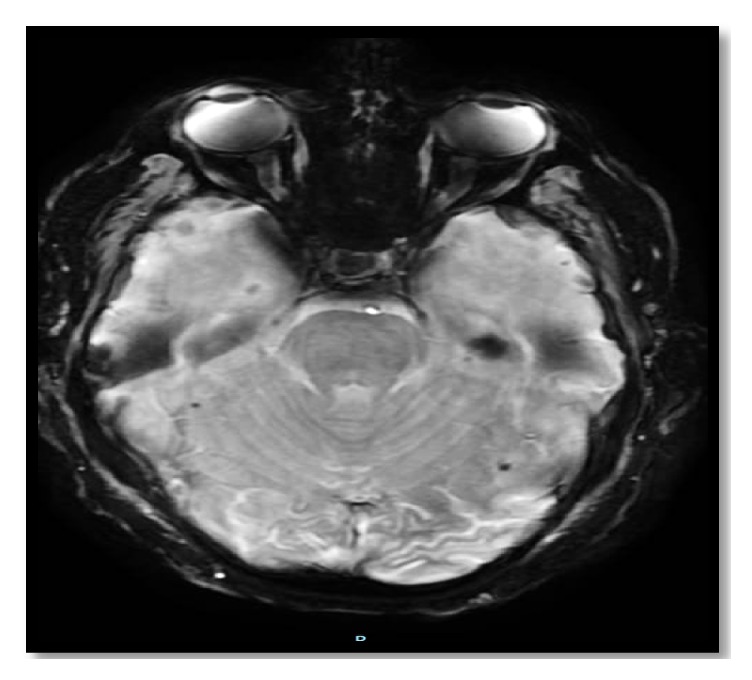
Periictal brain MRI SWAN image: showing gyriform hyperintense signal abnormality over bilateral occipital cortex. Abbreviations: MRI, magnetic resonance imaging; SWAN, susceptibility weighted angiography.

**Figure 6:**
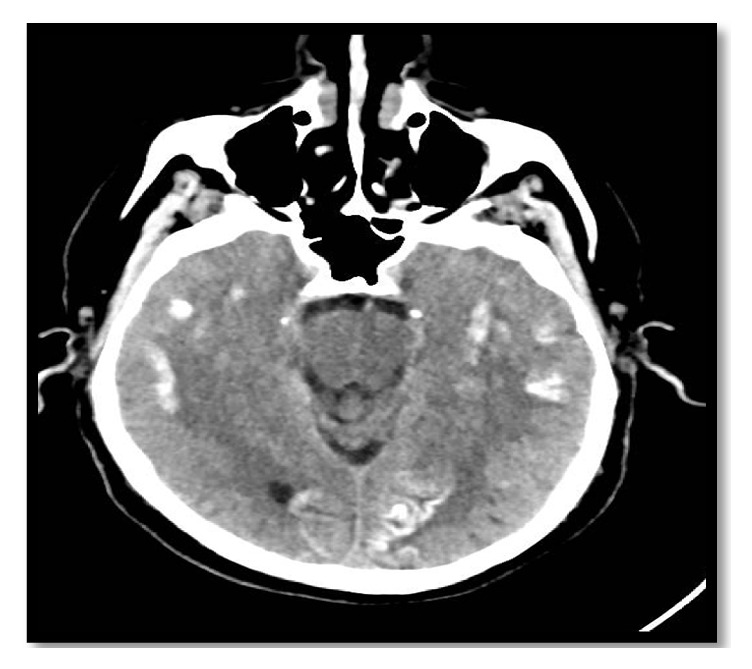
Postictal plain CT of the brain showing no interval abnormality compared to the CT of the brain upon presentation. Abbreviation: CT, computed tomography.

The computed tomography (CT) of the patient's brain showed bilateral scattered corticosubcortical parietooccipitotemporal calcification with no oedema or mass effect (Figure 1). The brain MRI with and without contrast showed diffusion-weighted imaging (DWI) restriction over bilateral occipital cortex (more so over the left side) in a gyriform pattern (Figure 2) with concordant area of apparent diffusion coefficient (ADC) hypointensity (Figure 3). Susceptibility weighted magnetic resonance sequences (susceptibility weighted imaging [SWI], susceptibility weighted angiography [SWAN]) demonstrated hyperintensity corresponding to the area of DWI restriction (Figure 5). T1-weighted imaging with contrast showed no contrast uptake (Figure 4), and we saw no oedema or mass effect. We found no cortical atrophy or any deep cerebral vein enlargement. A second brain CT after 1 month (Figure 6) showed no interval change compared to the initial CT and no evidence of residual changes observed in MRI (DWI, ADC, and SWI).

### 2.1. Differential Diagnosis

Bilateral cortical calcification has a distinct set of mimickers, and Sturge–Weber syndrome (SWS) is first among the possible differentials. Our patient's brain CT may indeed look identical to SWS. However, it is the constellation of clinical and radiological features that differentiate the two conditions. The important clinically distinguishing feature of SWS is our patient's normal cognition. Psychomotor retardation is seen in 50% of SWS cases [4], and our patient lacked facial nevus which is prevalent in most SWS cases (except SWS Roach classification type 2) [5]. Our patient also had no ocular disease, a presentation found in 77% of SWS cases. Radiologically, SWS has tram line or gyriform-only cortical calcification (and not subcortical, as in our patient). Bilateral calcification (as seen in our patient) occurs in only 25% of SWS patients [6]. Other radiological findings of SWS not seen in our patient are ipsilateral choroid plexus hypertrophy [7], enlarged transcortical (medullary) veins [8], ipsilateral cortical atrophy [9], enlargement of the ipsilateral ventricle, loss of volume of the ipsilateral cranial cavity [10], and pial enhancing angiomatous malformation (usually ipsilateral to the facial angioma). Therefore, many classic SWS features were not identified in our patient, distinguishing his condition from SWS.

Other conditions known to cause cortical calcification in a similar pattern are, namely, congenital folate malabsorption or the adverse effects associated with methotrexate and antifolate agents. There was no mention of the use of either of these agents. Congenital folate malabsorption would have an associated life-long history of symptoms of malabsorption [11] which is not apparent in our patient. Cortical laminar necrosis could account for such radiological presentation. However, this entity is conventionally thought to be a sequela of a remote traumatic brain injury, anoxic-ischaemic injury, or a metabolic insult that is not present in this patient's past history. Moreover, characteristically, cortical laminar necrosis has T1-weighted gyriform hyperintensity in MRI which was not noted in this patient. Although each of these conditions gives rise to calcified shadows in brain imaging, none are anti-TTG IgA-positive which is specific for the diagnosis of CD.

### 2.2. Treatment

Antiepileptic treatment with phenytoin and levetiracetam was commenced immediately upon the realisation that the patient has seizures. With the diagnosis of probable CEC, he was started on a strict gluten-free diet.

### 2.3. Outcome and Follow-Up Evaluation

The patient recovered remarkably and regularly submits to follow-up examinations at our neurology clinic. At his last visit (9 months from his presentation), we found no residual neurological deficit other than mild homonymous hemianopia. Currently, he is fully active, self-sufficient, and has not sustained any seizure for the past 9 months.

## 3. Discussion

CEC is a rare syndrome marked by CD, epilepsy, and bilateral calcifications in the occipital region [3]. From the time of its discovery, CEC syndrome has shown a predilection for Italian, Argentinian, and Spanish people [12]. However, a recent case series reported CEC syndrome in 3 Australian patients [13]. Reports of CEC also have emerged from South America [3], and this case represents the first from Saudi Arabia. Conventionally, patients with CD present with gastrointestinal signs and symptoms. Of note, CD may rarely present in silent or latent forms [14] characterized (in the absence of gastrointestinal symptoms) by other miscellaneous conditions like dermatitis herpetiformis, dental enamel defects autoimmune thyroiditis, anaemia, metabolic bone disease, coagulation disorders, infertility, psychiatric illness, and neurological symptoms [15]. The neurological features that are known to occur (around 20% of symptomatic CD) are varied and include myopathy, epilepsy, leukoencephalopathy, headache, gluten ataxia, and, rarely, CEC syndrome [16]. Our patient had no classical features of CD but had high antibody titre and mildly abnormal duodenal histology. Characteristic histopathological findings of CD were not identified in our case, which could be related to poor handling of biopsy specimen. However, it showed increased intraepithelial lymphocytosis with normal villous architecture (Marsh I lesion) which appears in up to 3% of duodenal biopsies [17]. Such findings may represent a wide range of conditions, namely, CD, bacterial overgrowth, nonsteroidal antiinflammatory drug damage, reaction to Helicobacter pylori infection, tropical sprue, and chronic inflammatory bowel disease [17]. Other than CD, none of these conditions would give rise to high anti-TTG. Therefore, it is plausible that these histopathological changes are markers of early CD.

The pathogenesis of CD has yet to be elucidated. However, CD may be due to an immune autoinflammatory reaction occurring in a genetically predisposed gluten-intolerant subject that originates at the jejunal mucosa and propagates to the lamina propria, culminating into the classic histopathological appearance of CD (i.e., crypt hyperplasia, jejunal villous atrophy with inflammatory infiltrate in the lamina propria). Furthermore, circulating activated T cells may cross the blood-brain barrier and be toxic to myelin or myelin-producing cells. This immune theory is substantiated by the fact that 95% and 5% of CD patients have HLA-DQ2 and HLA-DQ8 haplotypes, respectively [18]. These same haplotypes predispose patients to bilateral occipital calcifications and epilepsy [19]. One theory suggests that malabsorption develops into nutritional deficiency and reduced central nervous system folate levels, further inducing neurological dysfunction and calcifications in CEC syndrome patients [20, 21]. Many, on the contrary, theorize that folate is an unlikely interceder [3]. The mineralization (i.e., calcification) was recently proposed to be secondary to direct autoimmune effects from a specific antibody to neuronal transglutaminase isoenzyme [13, 22].

The epilepsy featured in CEC syndrome can be protean and may range from benign epilepsy to drug-resistant epilepsy, or even to epileptic encephalopathy [23]. Seizure onset usually occurs around the age of 6 [24] and is typically a partial seizure, either focal or complex [3]. In typical CEC syndrome, patients present with paroxysmal visual symptoms that precede the seizure episode (e.g., blurred vision, visual hallucinations, coloured dots, inability to focus [23], or even temporary blindness [25]). Such presentation is suggestive of seizure activity arising in the occipital region, which is also explained by the bilateral calcifications found on imaging. Our patient's history of “bumping into surrounding objects” and episodic loss of awareness seems epileptic and was later escalated to generalized subclinical and even clinical status epilepticus.

Brain imaging in CEC syndrome patients reveal calcification in subcortical and cortical areas of both occipital lobes without contrast enhancement and brain atrophy [3, 26]. Our patient showed calcification additionally in corticosubcortical areas of the temporoparietal lobe. The calcified lesion does not change much in size over time, but cases of newly appearing lesions have been described. Retrospectively, we believe the history of reported small dots in our patient's brain imaging at the time of minor head trauma was indicative of a calcified lesion. MRI brain abnormalities described in the literature depict changes suggestive of calcified lesion (i.e., T2 hypointensity and SWI hypointensity). However, changes attributed to periictal metabolic abnormality seen in our patient have not been reported. Recent reports indicate some specific signal alteration occurring in DWI and ADC sequences during periictal phase [27]. As observed in our patient, transient focal hyperintensity on DWI with a concordant reduction in ADC related to seizures is presumed to epitomize cellular swelling in the area of seizure onset as a result of cytotoxic oedema from seizure-induced excessive neuronal metabolic demand which is inadequately addressed by the available blood supply [28]. The other bewildering issue is the presence of SWI hyperintensity signal over the area believed to be showing periictal changes in DWI. The SWI hyperintense signal at this site is perplexing for us and warrants further exploration. As for management, dietary gluten restriction and folate supplementation have improved the epilepsy component of CEC syndrome in some patients [20, 29]; however, reports of difficult treatment and resistance have been described in the literature [30]. Initiating treatment earlier in young patients has a favourable outcome [20, 29]. Our patient showed a favourable response to a strict gluten-free diet and antiepileptic drug adherence.

In conclusion, our patient's episodic visual disturbance and confusion were likely a reflection of episodic occipital seizure or subclinical seizure that rendered transient signal abnormalities in MRI in this first-ever reported case of probable CEC syndrome in a Saudi patient.
